# Medication errors—an enduring problem for children and elderly patients

**DOI:** 10.3109/03009734.2012.659771

**Published:** 2012-08

**Authors:** Sergey Zakharov, Navratil Tomas, Daniela Pelclova

**Affiliations:** ^1^Health Law Centre, Third Faculty of Medicine, Charles University in Prague, Czech Republic; ^2^Toxicological Information Centre, Department of Occupational Medicine, First Faculty of Medicine, Charles University in Prague and General University Hospital, Prague, Czech Republic; ^3^Department of Biophysical Chemistry, J. Heyrovský Institute of Physical Chemistry of AS CR, v.v.i, Prague, Czech Republic

**Keywords:** Drug overdose, improper route of administration, medication errors, wrong drug administration

## Abstract

**Objective:**

To analyze the types and reasons of medication errors, committed by health care professionals, which led to toxicological consultations at the Czech Toxicological Information Centre (TIC).

**Methods:**

Inquiries arising from medication errors for 2000–2010 were extracted and evaluated from the database of the TIC, recording the consultations of poisonings due to drugs, household products, plants, and mushrooms.

**Results:**

From a total of 44,344 calls concerning pharmaceuticals, 215 (0.5%) were denoted by the caller as medication errors; 130 involved children (90 below 5 years of age) and 85 involved adults (30–60 years of age). The most common errors were: improper dosage (60.9%), wrong medication (19.3%), or erroneous route of administration (12.9%). The most frequent medication errors appeared using drugs affecting the nervous system (psycholeptics and antiepileptics), antibiotics, and drugs affecting the respiratory system. Nurses administering the drugs were responsible for 43.0%, physicians prescribing the drugs for 36.8%, and pharmacists dispensing the drugs for 20.2% of the errors. Of 25 patients with severe drug intoxications, 60.0% were children under 5 years of age treated with pharmaceuticals affecting the CNS, and 28.0% patients over 60 years of age with chronic application of theophylline, digoxin, or lithium.

**Conclusions:**

The trend in medication errors has remained relatively stable over the past 11 years. The analysis of medication errors shows two high-risk categories: children of less than 5 years of age, in whom the dose was not correctly adjusted, and elderly people with chronic medication and insufficient control of their medication level. Therefore, the measures for risk reduction should focus primarily on them.

## Introduction

Medication errors are an important cause of patient morbidity and mortality and have remained the focus of attention of health care quality experts for more than 10 years after the landmark publication of the American Institute of Medicine appeared (1–6). Unlike adverse drug reactions (ADRs) or adverse drug events (ADEs), they are caused by a mistake by the health care personnel.

Medical doctors then seek rapid qualified advice in the toxicological information center/poison control center to avert the risk of severe consequences of an inadvertent drug overdose or wrong pharmaceutical use and to treat the patient efficiently. Electronic data collected by toxicological information centers on telephone calls from health care professionals requesting a toxicological consultation are considered one of the relevant sources of information about the occurrence of medical malpractices related to the inappropriate use of medications in the process of health care provision (7–10).

The Czech Toxicological Information Centre (TIC) in Prague provides 24-h telephone consultations for both health care professionals and lay persons in the Czech Republic, a country with a population of 10 million, concerning the toxicity of a wide range of substances (chemicals, consumer goods, medicines, etc.), as well as on the diagnostics and treatment of acute poisonings.

To study the most common reasons, an analysis of the medication errors committed by health care professionals was conducted in the Czech TIC. The aim was to provide data that could help health care quality experts to develop further measures within the long-term strategy of the improvement of medical care quality and the reduction of risks associated with the so-called human factor in medicine.

## Methods

Of the total calls concerning the application of drugs in the studied period, medication errors committed by health care professionals were analyzed retrospectively from the electronic database of the TIC, which collects records for every toxicological inquiry. The completed questionnaires for the selected items and a full text description of the scenario, symptoms, and treatment already performed and further recommended are provided in detail. The individual situation is described that had led to the overdose or application of the pharmaceutical in an inappropriate way or of the wrong drug and clearly differentiates it from an ADR or ADE. The toxicological specialist of the TIC then makes the classification of the call as a medication error.

The data obtained were categorized into health care facility type, patient age group, medical staff involved, pharmaceutical administered, error type, and severity of symptoms. Numbers of calls in the different categories were then compared to find the more frequent types.

The health care facility type included in-patient departments of hospitals, out-patient medical facilities, and pharmacies. The category of medical staff involved physicians, nurses, and pharmacists. The age groups were: babies (0–1 year), children 1–5 years, children over 5 years (5–18 years), and adults under 45 years of age, adults over 45 years of age, and senior adults (older than 60 years). Drugs administered were classified according to the Anatomical Therapeutic Chemical (ATC) classification (11,12). Error type involved the dosing, route of administration, administration, or dispensation of the wrong medication, and to the wrong patient. The estimations of the drug doses were: non-toxic, minor toxic, moderate toxic, toxic (severity unknown), severe toxic, and unknown. Severity of symptoms of intoxication was classified according to the Poisoning Severity Score (13).

## Results

The TIC received 215 calls from health care professionals due to medication errors from 2000 to 2010, which represented 0.5% of all the calls concerning drugs (Table I).

**Table I.** Total number of calls to the Czech Toxicological Information Centre, concerning drugs and medication errors in 2000–2010.

	2000	2001	2002	2003	2004	2005	2006	2007	2008	2009	2010	Total
Total calls concerning drugs	3170	3607	3637	3446	3768	4086	4352	4901	4212	4181	4984	44344
Medication errors	20	21	22	16	22	19	20	18	17	16	24	215

A total of 61.9% of calls concerned medication errors in the in-patient departments of hospitals, 18.6% in the out-patient medical facilities, and 19.5% in the pharmacies.

**Figure 1.**
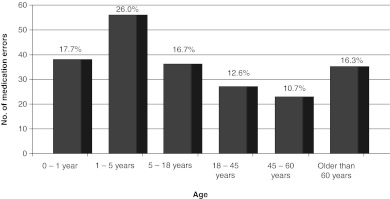
Age of patients affected by medication errors registered by the Czech Toxicological Information Centre from 2000 to 2010.

From the calls, 130 involved children, and 85 concerned adults. Of all of the patients with medication errors committed in their treatment, children constituted 60.4%, among them 43.7% were under 5 years of age. Among adults, 41.2% of the patients were older than 60 years (Figure 1).

**Figure 2.**
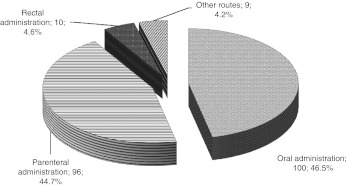
Routes of drug administration in medication errors registered by the Czech Toxicological Information Centre from 2000 to 2010 (counts and percentages are given).

The involvement of drug classes according to the Anatomical Therapeutic Chemical (ATC) classification in medication errors is shown in Table II. The most frequent medication errors involved drugs affecting the nervous system, especially psycholeptics (haloperidol, promethazine, lithium, etc.) and antiepileptics (valproates, phenytoin, lamotrigine, etc.). Second place was occupied by antibiotics; third place by drugs affecting the respiratory system, where acute and chronic overdoses of theophylline preparations constituted the major part. Further, the erroneous administrations of antiemetics and antinauseants (thiethylperazine, metoclopramide) represented a considerable percentage for drugs affecting the alimentary tract. The analysis of the routes of drug administration is shown in Figure 2.

**Table II.** The drug classes according to the Anatomical Therapeutic Chemical (ATC) classification involved in medication errors in the calls to the Czech Toxicological Information Centre in 2000–2010.

Drug class (ACT classification)	Total number of calls	Number of calls	Total percentage of the calls (%)	Percentage of the calls (%)
N (Nervous system):	54		25.1	
N01 (anesthetics)		4		1.9
N02 (analgesics)		2		0.9
N02A (opioids)		7		3.3
N03 (antiepileptics)		13		6.0
N05 (psycholeptics)		17		7.9
N05CA, N03AA (barbiturates)		5		2.3
N05BA, N03AE (benzodiazepines)		4		1.9
N06A, N03AF (tricyclic antidepressants, carbamazepine)		2		0.9
J (Anti-infectives for systemic use):	33		15.3	
J01 (Antibacterials for systemic use)		33		15.3
R (Respiratory system):	29		13.5	
R03 (drugs for obstructive airway diseases)		15		7.0
R05CA (expectorants)		5		2.2
R05CB (mucolytics)		4		1.9
R05D (cough suppressants)		1		0.5
R06 (antihistamines for systemic use)		4		1.9
A (Alimentary tract and metabolism):	24		11.2	
A02 (drugs for acid-related disorders)		1		0.5
A03 (drugs for functional gastrointestinal disorders)		1		0.5
A04 (antiemetics and antinauseants)		13		6.1
A07 (antidiarrheals, intestinal anti-infective agents)		2		0.9
A10 (drugs used in diabetes)		2		0.9
A11 (vitamins)		3		1.4
A12 (mineral supplements)		2		0.9
D (Dermatologicals):	21		9.8	
D06 (antibiotics and chemotherapeutics for dermatological use)		4		1.9
D08 (antiseptics and disinfectants)		15		7.0
D11 (other dermatological preparations)		2		0.9
C (Cardiovascular system):	10		4.7	
C01 (cardiac therapy)		5		2.3
C03 (diuretics)		1		0.5
C04 (peripheral vasodilators)		2		0.9
C07 (beta-blocking agents)		1		0.5
C08 (calcium channel blockers)		1		0.5
L (Antineoplastic and immunomodulating agents):	10		4.7	
L01 (antineoplastic agents)		7		3.3
L03 (immunostimulants)		1		0.5
L04 (immunosuppressants)		2		0.9
M (Musculo-skeletal system):	4		1.9	
M02 (anti-inflammatory and antirheumatic products)		3		1.4
M04 (antigout preparations)		1		0.5
B (Blood and blood-forming organs):	4		1.9	
B02 (antihemorrhagics)		1		0.5
B03 (antianemic preparations)		3		1.4
G (Genito-urinary system):	2		0.9	
G03 (sex hormones)		2		0.9
H (Systemic hormonal preparations):	2		0.8	
H02 (corticosteroids for systemic use)		2		0.8
S (Sensory organs): S01 (ophthalmologicals)	2	2	0.9	0.9
V (Various)	11	11	5.1	5.1
P (Antiparasitic products)	1	1	0.5	0.5
Combination of different drugs	8	8	3.7	3.7
Total	215		100.0	

**Figure 3.**
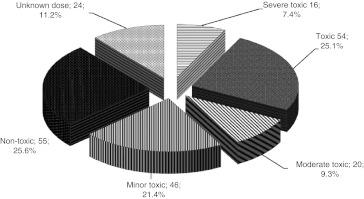
Estimation of doses of drugs administered to patients due to medication errors registered by the Czech Toxicological Information Centre from 2000 to 2010 (counts and percentages are given).

The estimation of the administered doses indicated that overdoses occurred in 63.4% of calls, including 7.4% with doses classified as severly toxic (Figure 3). In 44.2% of the inquiries, health care professionals called the TIC before the onset of the first signs and symptoms of intoxication.

**Figure 4.**
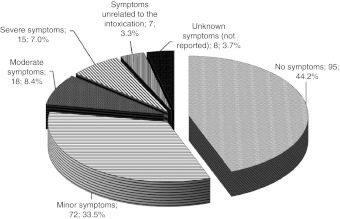
Symptoms of drug intoxication at the time of toxicological consultation provided due to medication errors registered by the Czech Toxicological Information Centre from 2000 to 2010 (counts and percentages are given).

An evaluation of the symptoms occurring at the time of the call is presented in Figure 4. Severe drug intoxications were 25 (11.6%); children under 5 years old were involved in 15 (60.0%) and senior adults in 7 (18.0%) of them. Forced diuresis (in 5 patients), extra-corporeal elimination method (in 19 patients), or specific antidotes (in 4 patients) were recommended in 28 (13.0%) calls.

The following case reports illustrate a number of severe intoxications in children and elderly adults:

*Case 1:* An 8-day-old newborn with neonatal seizures had received phenobarbital inj. i.v. repeatedly and fell into a coma. The phenobarbital blood level was in the lethal range, hemoperfusion was performed; however, the patient died.

*Case 2:* A 3-month-old baby had received a single i.v. injection of phenobarbital; the dose was four times higher than the maximum therapeutic dose. The child had been unconscious during the inquiry. Hemoperfusion was performed, and the child survived.

*Case 3:* A 4-month-old baby was given one dose of neostigmin inj. i.v.; the dose was ten times higher than the maximum therapeutic dose. The baby died from cardiac arrest.

*Case 4:* A 70-year-old female treated with theophylline orally, without blood level controls for several months, had been admitted to the hospital due to seizures and tachycardia, and lethal serum theophylline concentrations were recorded hemoperfusion was carried out and the patient survived.

*Case 5:* A 76-year-old male with chronic kidney failure hospitalized at the internal department had been treated with digoxin without proper dosage adjustment, so he developed severe bradycardia, hyperkalemia, and acidosis; hemoperfusion was performed, and the patient survived.

As to the categories of staff, nurses were involved in 43.0%, medical doctors in 36.8%, and pharmacists in 20.2% of medication errors (Table III).

**Table III.** Types of medication errors and medical staff involved according to the calls of health care workers to the Czech Toxicological Information Centre from 2000 to 2010.

Characteristics of medication errors	Number of errors			Percentage of the calls (%)		
	Total	Subtotal		Total	Subtotal	
Nurses' errors:	96			43.0		
Drug overdose, total:		38			17.0	
one-time			31			13.9
Repeated (chronic)			7			3.1
Wrong drug administration		29			13.0	
Wrong route of administration		21			9.4	
Administration to wrong patient		8			3.6	
Physicians' errors:	82			36.8		
Drug overdose, total:		60			26.9	
one-time			32			14.3
Repeated (chronic)			28			12.6
Wrong drug administration		14			6.3	
Wrong route of administration		8			3.6	
Pharmacists' errors:	45			20.2		
Improper (higher) dosage		38			17.0	
Wrong drug dispensation		7			3.2	
Total	223	223	-	100.0	100.0	-

Among the medication errors committed by nurses, the most frequent one was the wrong dose administration (39.6%), and one-time overdoses (32.3%) prevailed against repeated ones (7.3%). A significant part of nurses' errors (30.2%) consisted of cases of wrong drug administration, i.e. unintentional substitution of the prescribed preparation for another one. The medications inadvertently substituted were so-called ‘sound-alike’ medications (e.g. Diazepam/Dithiaden, Solutan/Solvolan, Isoptin/Isoprinosine, etc.) ones. The errors associated with the improper route of administration, such as an intravenous administration of intramuscular preparation (depot forms), occupied the third place in the category of nurses' errors (21.9%), and were followed by wrong-patient errors (8.3%).

A further breakdown of 82 medication errors committed by physicians indicated that the main part again related to drug overdoses (73.2%), either single (39.0%), or repeated ones (34.2%). The proportion of improper dose administration was almost two times higher than in nurses' medication errors. Second place was occupied by cases of wrong drug administration (17.1%), followed by wrong-route-of-administration cases (9.7%).

A review of 45 medication errors committed by pharmacists indicated that this category had been constituted by two groups. The first one (84.4%) was associated with providing a patient with drugs in a higher dosage than had been prescribed by a doctor. Pharmacists had taken no heed of the higher ‘adult’ strength of tablets or suppositories designated for children, inadvertently substituted syrups with more concentrated drops, or recommended improper dosage regimes based on miscalculations, etc. The other group (15.6%) concerned dispensing the wrong drugs, i.e. substitution of medications prescribed by a physician with another similar sounding and similar looking drug.

## Discussion

The analysis and interpretation of data obtained are characterized by several limitations. Primarily, physicians consult the TIC on a voluntary basis, and the number of errors registered does not reflect the actual quantity. Therefore, a significant number of occurrences that might be identified by other means, such as a retrospective study of hospital medical records, electronic mandatory and voluntary adverse event reporting systems, or direct observational methods, remain unseen. On the other hand, this approach allows one to concentrate on the serious adverse incidents that have compelled physicians to apply for ‘external assistance’. TIC considers the data supplied by the caller to be reliable. The calling physician in the difficult situation usually gives the exact data, as any misleading information could result in an inadequate treatment and endanger the patient.

The data obtained can be beneficial for health care quality experts in identifying the process, finding functional and structural ‘weak points’ predisposing to medical malpractices within the system of drug administration, and developing systemic measures aimed at the reduction of the risks of analogous errors in the future. The quantitative analysis of the types of medication errors indicated that the most common ones were improper dose administration, wrong drug administration, improper route of administration, and administration to the wrong patient. There was more than one error (several different errors) present within the same occurrence in some of the cases analyzed. The study supports the previous finding that pediatric patients are generally exposed to a higher risk of potentially dangerous medication errors than adults (14). For all three categories of health care professionals, applying an improper dose was the most frequent type of error.

Nursing was the number one category of medical disciplines involved in the medication errors. One of the probable reasons for this fact is that nurses spend up to 40% of their work time administering medications (15). Furthermore, drug administration is referred to as the ‘sharp edge’ in the medication-use process, because a great deal of preventable medication errors occurs at this ‘error-prone’ administration step (16).

It should be emphasized that, although the psychological nature of a medication error differs in the internal mechanism and external conditions, some common features could be identified. The internal mechanism of a medical doctor's error appears mainly realized at the cognitive level, manifesting in the erroneous or inadequate assessment of a patient's individual characteristics (e.g. age, weight, renal and hepatic functions) and drug kinetics (possibility of accumulation, interactions), insufficient monitoring of chronic medication, wrong decision-making or ‘memory lapse’ in urgent situations, etc. Therefore, systemic measures aimed at the prevention of the medication errors should include eliminating the stressogenic factors during the drug administration process and providing the doctor with timely and sufficient information on the actual state of the patient (standards of monitoring chronic medication) and the drugs administered (electronic databases of drug interactions).

The mechanism of the medication error committed by a nurse may unfold primarily at the perceptual or motor level. Pharmaceuticals with similar sounding names are commonly regarded as a kind of ‘high-risk group’ for wrong-drug-administration errors with possible serious consequences for the patient's health (17,18). The problems of drugs with similar appearance and similar sounding names, labeling, and packaging of the same-name medications in different forms (strengths) are associated with insufficient (inadequate) distinctive properties of perceptual information provided and deficient attention paid to its content. Errors at the motor level are committed within the routine highly repetitive actions characterized as ‘mechanical’ or ‘automatic’, owing to the lack of awareness, low mental involvement, or insufficient conscious control of motor actions. This mechanism is mainly responsible for so-called ‘wrong-route errors’, such as an improper route of administration or with the wrong patient, which has been reported internationally as a critical concern (19). Therefore, the problem of nurses' medication errors should be solved primarily in the plane of the improvement of the quality of perceptual information received by the nurses and enhancement of vigilance during routine procedures (dual control algorithms of task fulfillment).

Nearly one-fifth of calls to the TIC concerned pharmacists' errors within the process of providing a patient with drugs prescribed by an out-patient physician. It is alarming that such a mistake is often revealed only by the patient himself/herself. Therefore, the mechanism of quality control and error prevention is not sufficiently effective in these cases and requires the introduction of certain additional measures like an out-patient physician's early (probably at least telephonic) monitoring of the correctness of drug application in the error-prone cases of drug prescription.

The analysis of the severity of health consequences (outcomes) of medication errors revealed 25 cases of severe drug poisoning (11.6%), with 7 lethal ones (3.3%). In most instances, severe intoxications were caused by intravenous drug administration (18 cases). Generally, injectable medications are of particular concern due to the high likelihood for harm (20). According to Hatcher et al. 54.0% of potential adverse drug events and 61.0% of serious and life-threatening errors are associated with intravenous medications (21).

Concerning other routes of drug administration, severe poisonings following a one-time oral (rectal) administration of a pharmaceutical were more characteristic for children below 1 year of age than for the other age categories.

What is noteworthy is the fact that 60.0% of cases of severe drug intoxication involved children under 5 years of age. In these patients, intravenous phenobarbital overdoses accounted for four severe acute poisonings (including two lethal outcomes); in addition, one child's death was registered after the intravenous administration of neostigmine. The other ten cases of serious child intoxications were caused by overdoses of pharmaceuticals affecting the central nervous system (valproates, midazolam and ketamine).

Elderly adults, on the other hand, were more likely to develop symptoms of serious intoxication following chronic oral administration of drugs with cumulative toxicity, such as theophylline, digoxin, and lithium, due to insufficient monitoring of medication (control of the lab values) by the physician.

The recommendation of enhanced elimination methods and specific antidotes can be considered as one piece of evidence of the severity of the case of drug intoxication. Of course, these methods are rarely applicable because of the limited indications, which is also similar to the antidotes. Nevertheless, in this study, indications for hemodialysis, hemoperfusion, forced diuresis, or antidote administration had been fulfilled in every eighth case of medication error.

## Conclusion

The most frequently reported medication errors were: overdose, wrong drug, wrong patient, and improper route of administration. Physicians and nurses were involved almost equally often in the errors, with some prevalence of nurses owing to their part in the drug administration process.

Every fifth reported case of wrong regime of medication or wrong drug application was associated with improper recommendations or with drug dispensing errors that occurred in pharmacies. This fact is of significant importance and indicates that the mechanism of quality control and error prevention at this stage of health care delivery is not sufficiently effective.

The most serious consequences for patients' health were related to overdoses of drugs administered via the intravenous route. Children under 5 years of age treated with pharmaceuticals affecting the central nervous system were exposed to a higher risk of serious health problems and death due to a medication error. Thus, special attention and precautions are appropriate when administering phenobarbital, neostigmine, ketamine, and midazolam in pediatric practices. For the patients over 60 years of age, the implementation of additional measures aimed at diminishing health risks associated with chronic application of theophylline, digoxin, and lithium is also pertinent. Altogether, these two high-risk categories accounted for 88% of the severe drug poisonings reported due to a medication error.Thus, analysis and publication of medication errors are essential requirements for the development and implementation of a long-term strategy of health care quality improvement and the continuous reduction of risks associated with providing health care.
